# The TissueNet v.2 database: A quantitative view of protein-protein interactions across human tissues

**DOI:** 10.1093/nar/gkw1088

**Published:** 2016-11-28

**Authors:** Omer Basha, Ruth Barshir, Moran Sharon, Eugene Lerman, Binyamin F. Kirson, Idan Hekselman, Esti Yeger-Lotem

**Affiliations:** 1 Department of Clinical Biochemistry & Pharmacology, Faculty of Health Sciences, Ben-Gurion University of the Negev, Beer-Sheva 84105, Israel; 2 National Institute for Biotechnology in the Negev, Ben-Gurion University of the Negev, Beer-Sheva 84105, Israel

## Abstract

Knowledge of the molecular interactions of human proteins within tissues is important for identifying their tissue-specific roles and for shedding light on tissue phenotypes. However, many protein–protein interactions (PPIs) have no tissue-contexts. The TissueNet database bridges this gap by associating experimentally-identified PPIs with human tissues that were shown to express both pair-mates. Users can select a protein and a tissue, and obtain a network view of the query protein and its tissue-associated PPIs. TissueNet v.2 is an updated version of the TissueNet database previously featured in NAR. It includes over 40 human tissues profiled via RNA-sequencing or protein-based assays. Users can select their preferred expression data source and interactively set the expression threshold for determining tissue-association. The output of TissueNet v.2 emphasizes qualitative and quantitative features of query proteins and their PPIs. The tissue-specificity view highlights tissue-specific and globally-expressed proteins, and the quantitative view highlights proteins that were differentially expressed in the selected tissue relative to all other tissues. Together, these views allow users to quickly assess the unique versus global functionality of query proteins. Thus, TissueNet v.2 offers an extensive, quantitative and user-friendly interface to study the roles of human proteins across tissues. TissueNet v.2 is available at http://netbio.bgu.ac.il/tissuenet.

## INTRODUCTION

Proteins act through interactions with other molecules, and these interactions define their functions and their cellular roles in health and disease (1–3). Owing to their importance, many efforts have been invested in experimental mapping of physical interactions between proteins. In human, which is the focus of TissueNet, over 240 000 protein–protein interactions (PPIs) between more than 20 000 human proteins have been reported to date (4). These PPIs were detected by various experimental methods, and their records are available through several public databases.

Unlike unicellular organisms such as yeast, the human body is composed of many tissues and cell types, each expressing a distinct set of genes and proteins (e.g. (5–8)). Consequently, human proteins have different interaction partners across tissues and cell types (9,10). While this information is important for understanding the different functions of proteins across tissues, a tissue-sensitive view of PPIs is not readily available (for brevity ‘tissues’ also stands for cell types). Commonly applied PPI detection methods, such as protein arrays and yeast-two-hybrid, detect PPIs *in-vitro* or outside human cells. Other methods, like affinity-based assays, are typically carried in a single condition and not repeatedly across tissues (3,11).

A common approach for associating PPIs with tissues is by considering tissue expression data, such that PPIs involving lowly expressed or undetectable proteins are penalized or eliminated from the tissue view (e.g. (9,12,13)). The value of the resulting tissue-sensitive interaction networks (interactomes) was demonstrated in several applications, where tissue interactomes were shown to outperform the global, unfiltered interactome, in prioritizing disease genes (12–16) or to illuminate the molecular basis of tissue-selective hereditary diseases (17).

TissueNet was among the first databases that enabled users to obtain tissue-sensitive views of PPIs (18). By integrating gene and protein expression profiles of human tissues into a unified expression dataset, TissueNet provided extensive views into 16 main human tissues. Users could query TissueNet by using a protein and retrieve a network view of its PPI partners per tissue, or by using a PPI and retrieve the tissues expressing both pair mates. Importantly, in the output network TissueNet highlighted proteins that were tissue-specific or globally expressed, and by this, offered an intuitive, comparative view of tissue-associated PPIs.

Since the publication of TissueNet (18), additional databases that offer tissue-sensitive interactomes were developed, including GIANT (15), SPECTRA (19), HIPPIE (20) and IID (21). In most databases, whether relying on a single expression dataset (20) or consolidating multiple sources (15,18,21), tissue-associations are predetermined and the user cannot fine-tune the expression threshold for association, or explore different thresholds. Some databases support comparative analysis by enabling the user to select multiple tissues in a single query. For example, the output of IID (21) is a table of PPIs and their tissue-associations, with no network representation. The output of GIANT (15) includes a network view for each selected tissue. The output of SPECTRA (19) is a single network view, with distinct protein and edge colors representing distinct tissues. However, none of these output formats is scalable and takes into account the tens of different tissues that have been profiled to date.

**Figure 1.**
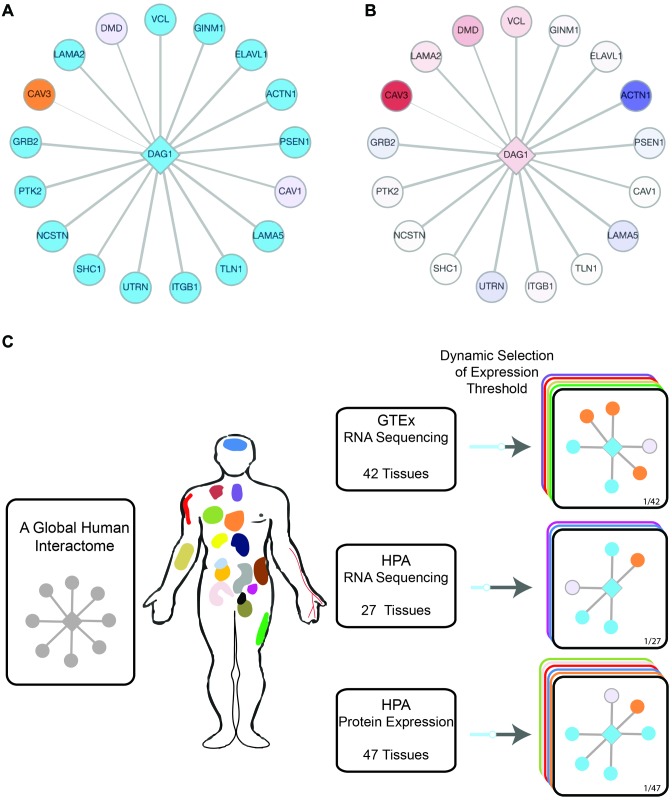
TissueNet v.2 output views and query flow chart. (**A**) The tissue-specificity view of the query protein DAG1 in muscle highlights its muscle-specific and globally expressed PPI partners. The DAG1 protein appears as a diamond-shaped node. Orange nodes denote muscle-associated proteins that were expressed in at most 20% of GTEx tissues, and blue nodes denote muscle-associated proteins that were expressed in at least 80% of GTEx tissues. (**B**) The quantitative view of the query protein DAG1 in muscle highlights its PPI partners that were significantly up- or down-regulated in muscle relative to other tissues. Node colors range from blue to red to denote down-regulated and up-regulated genes, respectively. White nodes denote genes with insignificant change in expression. This quantitative view is obtained directly from the tissue-specificity view by a toggle button. (**C**) A flowchart describing the integrative framework of TissueNet v.2. TissueNet analysis starts with a consolidated set of experimentally detected human PPIs. In each query, the user selects one of three data sources of tissue expression profiles, and can set the expression threshold for tissue-association. The output network view shows the query protein and its PPI partners that were expressed in the selected tissue at the threshold level or above.

The TissueNet database shows query proteins and their interactions in the selected tissue, using a network view that immediately compares this tissue to all other tissues by highlighting tissue-specific and globally expressed proteins (Figure 1A). As we previously showed, this comparison is key in studying the tissue-specific effects of disease proteins (17). TissueNet v.2 is an enhanced version of TissueNet that includes significant data expansion, increased usability, and provides a new quantitative view of the query protein and its tissue-associated interactions.

## ENHANCEMENTS AND NEW FEATURES IN TISSUENET v.2

TissueNet v.2 builds on the huge increment in the underlying data that opened the door for new features that were not possible with previous data. In addition to a 3-fold increase in PPI data, sources for tissue expression profiles of unprecedented scale became available. With TissueNet v.2, users have the flexibility to select the expression source and to set the expression threshold for tissue-associations interactively. By this, they can change dynamically the resulting network and fine-tune the tissue-specificity view of the presented proteins. The scale of the data allowed us to carry differential expression analysis for each tissue relative to all other tissues. By toggling a button, users can switch to a differential view of the same output network, and study quickly which interaction partners were up- or down- regulated in that tissue, and which were expressed similarly across tissues (Figure 1B). Below we describe in detail the increase in data and the new functionality of TissueNet v.2 that supports comparative and quantitative views of protein interaction sub-networks across tissues.

### New data incorporated into TissueNet v.2

TissueNet synergizes between large-scale data of human PPIs and expression profiles of tens of human tissues, to create an extensive database of tissue-associated PPIs. To this end, we gathered PPIs from four major PPI databases, BioGrid (4), IntAct (22), MINT (23) and DIP (24), and consolidated them by using the MyProteinNet web-server (25). This resulted in a global human interactome that contained 243 706 PPIs between 17 283 human proteins. The usage of MyProteinNet guaranteed that PPIs that were not detected by established experimental methods were excluded.

Since TissueNet was published (18), tissue expression profiles became available at unprecedented scale and quality. We extracted RNA-sequencing profiles from two leading sources: the Genome-Tissue Expression (GTEx) consortium (5) and the Human Protein Atlas (HPA) (6). From GTEx we included 421 samples from 42 tissues, and from HPA we included 192 samples from 29 tissues (see Methods). We associated between genes and their proteins products. To complement the RNA-sequencing data, we also extracted from HPA protein expression profiles based on antibody staining, which included over 14 000 proteins in 83 samples from 47 human tissues.

### Enhanced user flexibility

TissueNet v.2 offers users the ability to select the expression source by which to associate PPIs to tissues, and to set the expression threshold for the association (Figure 1C). The resulting network view shows the query protein with its PPI partners that surpassed the threshold in the selected tissue, colored by their tissue-specificity. A sliding bar allows users to repeatedly change the threshold and obtain an adjusted network. Another menu allows users to toggle between different tissues. The output menu also includes information about the expression levels of the presented proteins across tissues, their gene ontology (GO) annotations, and their PPI detection methods.

### A new quantitative view into tissue interactome differences

The extensive RNA-sequencing data provides a rich quantitative view of tissue expression that was previously unavailable. Specifically, it allows for identifying proteins that are differentially expressed in the selected tissue relative to other tissues. For this, we carried differential expression analysis per tissue (see Methods), and made it available to users through the quantitative view toggle button. By selecting this view, the output network is colored by the expression fold-change of the genes in the selected tissue, allowing users to immediately identify components that are up- or down-regulated in that tissue (Figure 1B).

## SUMMARY

The TissueNet v.2 database provides tissue-associated PPIs for tens of human tissues by integrating data of PPIs with data of gene and protein expression according to user-defined parameters. The output of TissueNet v.2 highlights qualitative and quantitative features of the PPI sub-networks that differentiate the selected tissue from other human tissues. By this, TissueNet v.2 offers a powerful means for illuminating general and tissue-specific protein functions, processes and phenotypes. Its scalable functionality and user-friendly interface can accommodate new data of additional tissues and cell types as they become available to increase precision and coverage of the database even further. With the increasing density and coverage of human PPIs, analysis tools that provide meaningful views into these huge amounts of data will become even more important in basic and applied research into human phenotypes and diseases.

## MATERIALS AND METHODS

### Expression data sources

Tissue expression profiles were obtained from GTEx (5) and HPA (6). From GTEx we gathered RNA-sequencing raw counts for all samples that were denoted with traumatic injury as the cause of death, resulting in 421 samples of 42 tissues. From HPA we gathered paired-end 100-bp raw RNA-sequencing reads for 192 samples of 29 tissues (ArrayExpress accession number: E-MTAB-2836). To convert raw reads to raw counts, we first trimmed them with Trimmomatic to remove adapter sequences and low-quality ends (parameters: illuminaclip slidingwindow:4:15 minlen:36). We aligned the trimmed reads to the GRCh38 assembly of the human genome using STAR version 2.3.0 with default parameters (26). Gene annotation was according to the genecode.v21.annotation.gtf file. Raw reads per gene that aligned to the reference genome uniquely were counted using HTSeq-count. Raw counts were normalized for each data source using the TMM method by the edgeR package (27), to obtain the same library size for every sample. Genes with less than 10 counts in all samples were removed before normalization. The normalized count for a gene in a tissue was set to its median normalized count in the corresponding tissue samples. We extracted data of protein expression for 85 samples from HPA (6). Samples taken from main tissues were united by associating each protein with its highest measured level (Supplementary Table S1).

### Differential expression analysis

Differential expression analysis was applied to GTEx tissues with at least five samples and to HPA tissues with at least three samples. In each sample, we transformed RNA-sequencing normalized counts using the VOOM method (28), and calculated differential expression using a linear model in the R-package Limma (29). Specifically, we compared all samples of the same tissue to all other samples in that data source. Only genes with FDR adjusted *P*-values <0.05 were considered to be differentially expressed and were colored according to their log_2_ fold-change values.

### Protein–protein interactions data

Human PPIs were downloaded from BioGrid (4), DIP (24), MINT (23) and IntAct (22), using the MyProteinNet web-server (25). The usage of MyProteinNet ensured that only PPIs detected by established methods for detection of physical interactions were considered, and resulted in a global interactome that contained a subset of the PPIs recorded in the different databases. PPI data will be updated every three months.

### Implementation

The TissueNet server was implemented in Python, using the Flask framework, with data stored on a MySQL database. The website client was programmed using the ReactJS framework and designed with Semantic-UI CSS. The network view is displayed by the Cytoscape.js plugin (30). The website supports all major browsers. Recommended viewing resolution is 1440×900 and above.

### Network view coloring

Network coloring is dynamic and depends on the data source and on the user-selected threshold on expression levels, as measured by normalized read counts. Only proteins whose expression level is not below the expression threshold are presented in the output network, and their tissue-specificity is also computed dynamically based on the same threshold. Since GTEx contains samples from tissues with multiple regions (e.g. 11 brain sub-regions), to compute tissue-specificity we grouped different regions of the same tissues as detailed in Supplementary Table S2.

### Download

The TissueNet database is available for download under the permissive Creative Commons license. Tissue interactomes were computed for each RNA-sequencing data source using a threshold of 8 normalized counts, and for HPA protein using a threshold of low expression. The distributions of proteins and PPIs by number of associated tissues were bimodal and similar across data sources (Supplementary Figure S1). Download data is versioned by numbered database builds and by global interactome build dates. The download page offers the user the ability to download data separately for each data source.
